# Deep learning-based gene selection in comprehensive gene analysis in pancreatic cancer

**DOI:** 10.1038/s41598-021-95969-6

**Published:** 2021-08-13

**Authors:** Yasukuni Mori, Hajime Yokota, Isamu Hoshino, Yosuke Iwatate, Kohei Wakamatsu, Takashi Uno, Hiroki Suyari

**Affiliations:** 1 Graduate School of Engineering, Chiba University, 1-33 Yayoi-cho, Inage-ku, Chiba-shi, Chiba 263-8522 Japan; 2 Department of Diagnostic Radiology and Radiation Oncology, Graduate School of Medicine, Chiba University, 1-8-1 Inohana, Chuo-ku, Chiba-shi, Chiba 260-8670 Japan; 3 Division of Gastroenterological Surgery, Chiba Cancer Center, 666-2 Nitona-cho, Chuo-ku, Chiba-shi Chiba, 260-8717 Japan; 4 Division of Hepato-Biliary-Pancreatic Surgery, Chiba Cancer Center, 666-2 Nitona-cho, Chuo-ku, Chiba-shi, Chiba 260-8717 Japan; 5 Media Data Tech Studio, CyberAgent, Inc., 13F Akihabara Daibiru, 1-18-13 Sotokanda, Chiyoda-ku, Tokyo 101-0021 Japan

**Keywords:** Microarray analysis, Machine learning

## Abstract

The selection of genes that are important for obtaining gene expression data is challenging. Here, we developed a deep learning-based feature selection method suitable for gene selection. Our novel deep learning model includes an additional feature-selection layer. After model training, the units in this layer with high weights correspond to the genes that worked effectively in the processing of the networks. Cancer tissue samples and adjacent normal pancreatic tissue samples were collected from 13 patients with pancreatic ductal adenocarcinoma during surgery and subsequently frozen. After processing, gene expression data were extracted from the specimens using RNA sequencing. Task 1 for the model training was to discriminate between cancerous and normal pancreatic tissue in six patients. Task 2 was to discriminate between patients with pancreatic cancer (n = 13) who survived for more than one year after surgery. The most frequently selected genes were *ACACB*, *ADAMTS6*, *NCAM1*, and *CADPS* in Task 1, and *CD1D*, *PLA2G16*, *DACH1*, and *SOWAHA* in Task 2. According to The Cancer Genome Atlas dataset, these genes are all prognostic factors for pancreatic cancer. Thus, the feasibility of using our deep learning-based method for the selection of genes associated with pancreatic cancer development and prognosis was confirmed.

## Introduction

Deep learning techniques have produced impressive results in many fields, including image classification, image generation, automated driving, and even board gameplay^1–6^. A great deal of research is now being conducted to apply the superior inference performance of deep learning to the field of medicine^7,8^. In deep learning, a large amount of data is required for training the model in order to extract the features that are useful for inference. In particular, in the field of image recognition, it is common to use convolutional layers that incorporate convolutional operations to capture local patterns in an image and emphasize specific features of the image. By layering multiple convolutional layers, complex higher-order features can be extracted, and image features for classification and inference with high accuracy can be extracted automatically. Therefore, deep learning has a very high proficiency for medical image processing, and various applications have been reported.

However, unlike images, the gene expression data used in this study are table-type numerical data that represent the expression state of each gene and are characterized by the fact that many features (genes) are interrelated. When analyzing such table-type numerical data using deep learning, it is necessary to consider all possible interactions between the input features. This is very different to processes like convolutional operations that only consider the interactions taking place around the pixel of interest, such as those in images. This means that we must consider the global interaction of all features using dense layers when analyzing such table-type numerical data. By combining these dense layers multiple times, we expect to be able to extract higher-order features that are effective for inferences.

In recent years, there has been a great deal of research on the interpretability of machine learning models^9–11^. For typical machine learning models such as logistic regression, random forests, and gradient boosting, we can interpret the basis for their inferences to some extent. On the other hand, it is generally difficult to reason out the inference results of neural networks. This is because neural networks repeatedly perform many layers of sum-of-product computation and nonlinear transformation on the input data in order to output predictions. In image processing, techniques such as Grad-CAM^12^ can be used to visualize the parts of the image the model has focused on to make inferences, thereby helping us to interpret the nature of deep learning. When using a neural network with multiple fully connected dense layers in table-type numerical data, millions of operations, or even tens of millions of operations, can be performed. Although understanding the exact flow of conversions that turns input data to output prediction is practically hard in a such process, the input features that affected the inference result by using model-independent methods such as Permutation Feature Importance^13^ and SHAP^14^ can be evaluated. However, the applicability of these methods to data with a low number of samples, more than 10,000 features, and complex interactions between the features is limited.

Current medical practices and healthcare industry obligations require the involvement of personalized medicine to be able to provide more appropriate treatment^15^. One health area where personalized medicine may be of significant benefit is cancer treatment. Pancreatic cancer is a malignant tumor with a poor prognosis. Surgery and multidisciplinary treatments, such as anticancer drug therapy and radiotherapy, are important for ensuring favorable pancreatic cancer outcomes. However, the optimal treatment method may differ for each patient. To practically realize personalized medicine, genes significantly associated with a cancer subtype need to be identified to stratify patients based on gene expression data^16–19^. However, the identification of important genes from complex gene data sets comprising tens of thousands of expression statuses is a considerable challenge. Various methods based on machine learning have been proposed for the feature selection of gene expression data, but there is no consensus on a standard method to be used^20–23^.

Feature selection based on machine learning can be categorized into the filter, wrapper, and embedding methods. The filter method, also known as feature ranking, is a method that quantifies and evaluates each feature according to a certain index. While it is computationally inexpensive, it is difficult to take into account the interrelationships among the multiple features and to evaluate them numerically. The wrapper method takes into account the interrelationships among the features by dynamically changing the feature subset to be evaluated, but the computational cost tends to be high because the machine learning model is trained each time the feature subset is evaluated. The embedding method is one where feature selection is done simultaneously with the training of the machine learning model, and it allows for the elucidation of the interrelationships among the features at reduced computational cost. However, depending on the machine learning model used, some problems can occur, such as over-fitting and difficulty in interpreting the results. Thus, each method has its own merits and demerits.

Particularly in the case of gene expression data, where the number of samples is small and the number of features (genes) is very large, it is common for the results to be completely different for each method. In addition, since the behaviors and functions of all genes are not yet known, it is useful to evaluate the results comprehensively from various perspectives using a wide variety of feature selection methods in order to make a truly appropriate gene selection. Therefore, the main purpose of this study was to propose a methodology of feature selection based on deep learning, which includes a feature selection layer that can evaluate the importance of features in a deep learning model, and to show the feasibility of a new methodology for gene selection that has not been used before.

## Results

### Patient background

Patient backgrounds are summarized in Table 1. Significant differences in CA19-9 levels, overall survival (OS), and disease-free survival (DFS) were observed between the better and worse prognosis groups. No significant differences were observed in the pathological variables.

**Table 1:** Clinical background for pancreatic cancer.

	All	Better prognosis	Worse prognosis	*p*-value
**All**	13	6	7	
**Sex**				
Male	8	5	3	
Female	5	1	4	
**Age**	64 (48–76)	66 (64–76)	63 (48–72)	0.352
**Overall survival (day)**	299 (143–1428)	942 (323–1428)	214 (143–299)	0.008
**Disease free survival (day)**	190 (77–1429)	696.5 (323–1429)	104 (77–190)	< 0.001
**Preoperative CEA**	3.4 (1.3–28.5)	3.75 (1.3–28.5)	3.1 (2.1–14.0)	0.214
**Preoperative CA19-9**	412.7 (2.0–32951.5)	19.3 (2.0–10447.0)	2124.5 (143.1–32951.5)	0.038
**Operation time (min)**	383 (212–469)	316.5 (253–403)	413 (212–469)	0.225
**Operation blood loss (ml)**	600 (110–1570)	687.5 (330–1570)	590 (110–900)	0.520
**Operation type**				
Pancreatoduodenectomy	9	3	6	
Distal pancreatectomy	4	3	1	0.196
**Cytology**				
Negative	11	5	6	
Positive	2	1	1	0.538
**Margin status**				
R0	12	6	6	
R1	1	0	1	
R2	0	0	0	0.538
**Differenciation**				
Well	1	0	1	
Moderate	9	4	5	
Poor	3	2	1	0.220
**Interstitium type**				
Int	11	5	6	
Med	1	1	0	
Sci	1	0	1	0.269
**Lympathic invasion**				
Negative	3	1	2	
Positive	10	5	5	0.441
**Vascular invasion**				
Negative	0	0	0	
Positive	13	6	7	1.000
**Neural invasion**				
Negative	1	1	0	
Positive	12	5	7	0.462
**Lymph node metastasis**				
Negative	1	1	0	
Positive	12	5	7	0.461
**p max diameter (cm)**	4.3 (1.5–8.5)	3.55 (1.5–8.5)	4.5 (3.6–4.5)	0.423
**pT (UICC) 8th**				
T1	1	1	0	
T2	4	3	1	
T3	8	2	6	0.065
**pStage (UICC 8th)**				
IA	0	0	0	
IB	1	1	0	
IIA	0	0	0	
IIB	3	2	1	
III	9	3	6	0.167

### Experiment tasks

In this study, we experimented with the selection of important genes over two separate tasks. The first task was to discriminate between pancreatic cancer and normal pancreatic tissue (Task 1). The second task was to discriminate patients with pancreatic cancer who survived for more than one year after surgery (Task 2). Task 1 used gene expression data of pancreatic cancer and normal tissues sampled from six patients. Task 2 used gene expression data obtained from 13 pancreatic cancer tissues (6, better prognosis; 7, worse prognosis). The selected genes from Tasks 1 and 2 were validated using the Cancer Genome Atlas dataset (TCGA: https://www.cancer.gov/about-nci/organization/ccg/research/structural-genomics/tcga).

### Results of gene selection

The top 25 genes and the number of times they were ranked in the top *N* among *K* trials of Task 1 and Task 2 are shown in Fig. 3. The number of trials was set to $K=1000$ in the experiments. Figure 3a and b show the results of Task 1 with $N=10$ and $N=50$, respectively, and Fig. 3c and d show the results of Task 2. The top 10 genes, representing the genes with the largest contribution to the proposed method, are shown in Table 2.

**Figure 1:**
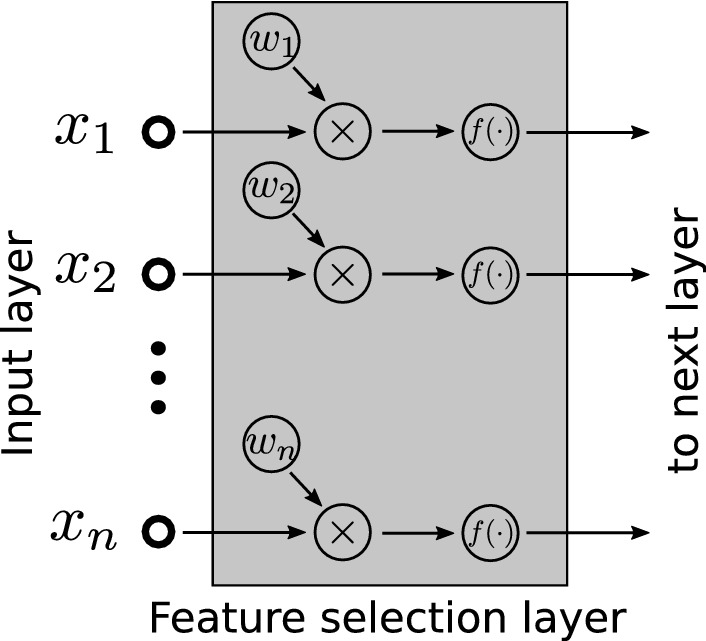
The network structure for the feature selection layer.

**Figure 2:**
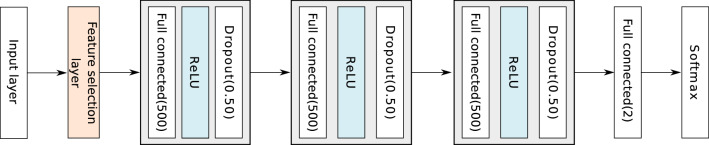
The network structure for gene selection used in Task 1 and Task 2.

**Table 2:** Results of gene selection for Task 1 and Task 2.

	Task 1		Task 2	
# of ranking	$N=10$	$N=50$	$N=10$	$N=50$
1	*ACACB*	*ACACB*	*CD1D*	*PLA2G16*
2	*ADAMTS6*	*ADAMTS6*	*PLA2G16*	*DACH1*
3	*NCAM1*	*CADPS*	*SOWAHA*	*CD1D*
4	*RGS1*	*NCAM1*	*DACH1*	*SOWAHA*
5	*CADPS*	*RAB3IP*	*TBC1D8*	*TBC1D8*
6	*RAB3IP*	*FAM107A*	*KIAA1217*	*KIAA1217*
7	*SLC4A4*	*TNXA*	*ITGB8*	*ITGB8*
8	*CCL28*	*LPCAT2*	*ZBTB46*	*CCDC142*
9	*FAM107A*	*RGS1*	*CCDC142*	*SLC16A7*
10	*TNXA*	*SLC4A4*	*SLC16A7*	*ZBTB46*

In each task, the genes selected with $N=10$ and $N=50$ were ranked almost the same. In Task 1, 9 out of 10 genes were the same. While the top two ranked genes in $N=10$ and $N=50$ were identical for Task 1 (*ACACB* and *ADAMTS6*), the third-ranked genes were different ($N=10$, *NCAM1*; $N=50$, *CADPS*). In Task 2, *CD1D* and *PLA2G16* were selected within the top three for both $N=10$ and $N=50$. While *CD1D* was selected first for $N=10$ and third for $N=50$, *PLA2G16* was ranked second for $N=10$ and first for $N=50$. *SOWAHA* was chosen in the third place for $N=10$, and *DACH1* in the second place for $N=50$. We also applied this method to TCGA data for gene selection, but the results were different to the results obtained using our data. This may be due to the small number of sample data we used, and the differences between our methods of data preparation and standardization.

### Survival analysis

Survival analyses using the Cancer Genome Atlas (TCGA: https://www.cancer.gov/about-nci/organization/ccg/research/structural-genomics/tcga)) data for all genes in the top 10 ranking for each task demonstrated the significance of 6 out of the 10 genes in Task 1 (both $N=10$ and $N=50$) and 9 out of the 10 genes in Task 2. The Kaplan-Meier plots of the four genes selected in the top three positions in Task 1 and Task 2 are shown in Figs. 4 and 5, respectively. The genes selected using this method were significantly associated with the prognosis of pancreatic cancer.

**Figure 3:**
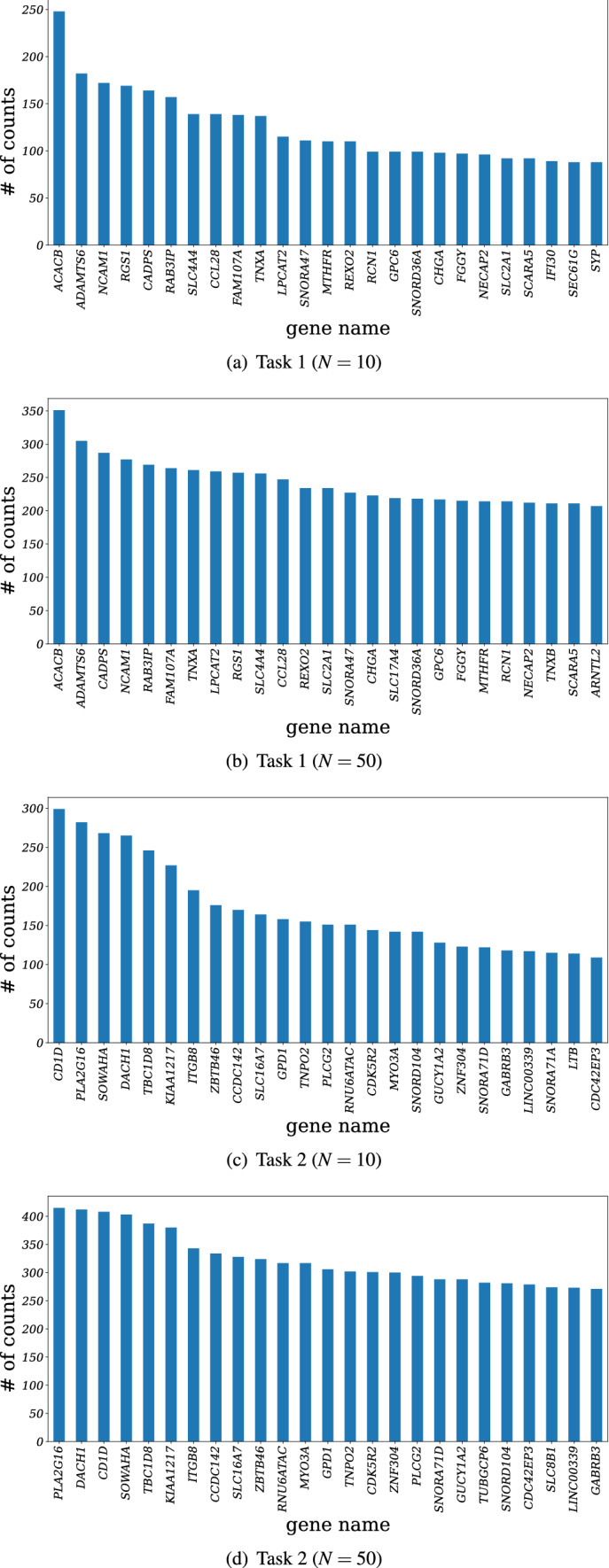
The number of counts of the top 25 genes ranked in the top 10 and 50 positions during 1000 trials.

**Figure 4:**
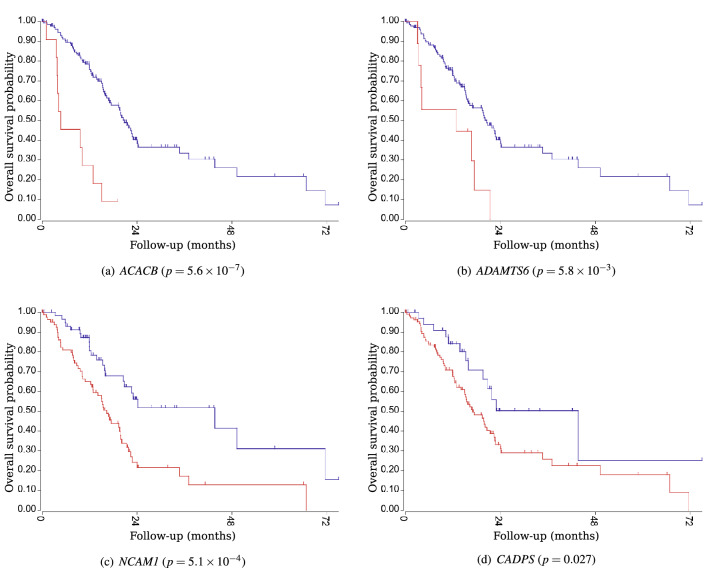
Kaplan–Meier plots of overall survival for the top three genes in Task 1. The top three genes were *ACACB*, *ADAMTS6*, and *NCAM1* at $N=10$, and *ACACB*, *ADAMTS6*, and *CADPS* at $N=50$. These genes were significant prognosis predictors.

**Figure 5:**
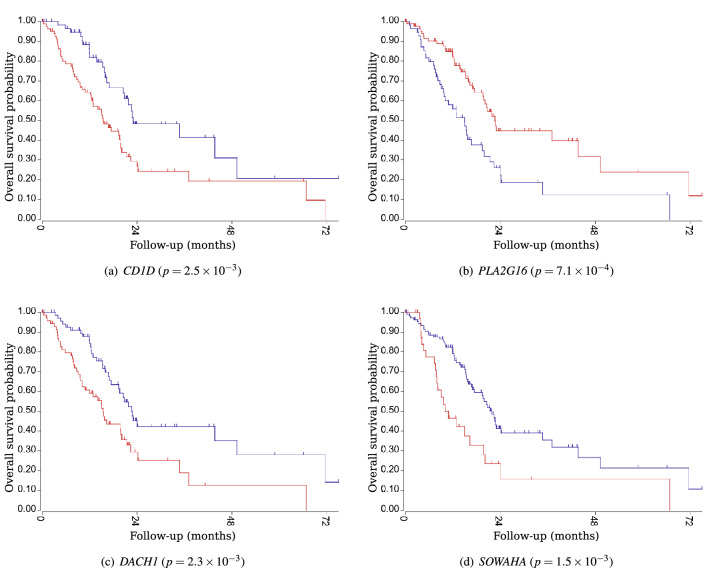
Kaplan meier plots for the top three genes in Task 2. The top three genes were *CD1D*, *PLA2G16*, and *SOWAHA* at $N=10$, and *PLA2G16*, *DACH1*, and *CD1D* at $N=50$. These genes were significant prognosis predictors.

## Discussions

In this study, we adapted our proposed deep learning model with a feature selection function for pancreatic genetic data. Important genes were selected in two separate classification tasks: pancreatic cancer vs. normal tissue; and better prognosis vs. worse prognosis. The selected genes were related to the prognosis of pancreatic cancer when evaluating TCGA dataset as an external data source.

The number of genes that were considered to be significant in TCGA dataset was higher in Task 2 than in Task 1 (9 of 10 vs. 6 of 10). Task 1 involved the selection of genes for effective binary classification between pancreatic cancer tissue and normal pancreatic tissue. Task 2 involved a binary classification between better prognosis and worse prognosis. Because Task 2 directly searched for prognostic determinants, it was unsurprising that the survival analysis in TCGA dataset showed more differences in Task 2 than in Task 1.

*ACACB* encodes acetyl-CoA carboxylase (ACC) $\beta$, a rate-limiting enzyme in fatty acid synthesis (where it plays a role in the regulation of fatty acid oxidation). The effects of ACC $\beta$ inhibition on the survival of pancreatic cancer cells have previously been investigated^24^. ACC $\beta$ induces apoptosis in some pancreatic cancer cell lines, suggesting that the use of ACC $\beta$ inhibitors may represent a new therapeutic strategy. *CD1D* (the top-ranked gene in Task 2) encodes CD1d, a transmembrane glycoprotein structurally related to major histocompatibility complex (MHC) proteins that activate natural killer T cells (NKT cells)^25^. The high ranking of *CD1D* may reflect the level of immunity it possesses against tumors. Furthermore, the other ranked genes are also likely to be strongly involved in the progression or control of pancreatic cancer, although further studies are needed to elucidate the details.

Feature selection is often used as a preprocessing step for machine learning and usually improves model performance and learning speed. If there are *k* features in total, the number of all combinations of features is $2^k-1$. Therefore, if *k* is sufficiently large, it is practically impossible to evaluate all feature subsets in a full search, and a feature selection algorithm is used to select $m(\ll k)$ features. Nevertheless, the evaluation of gene expression data with more than 10,000 features (as used in this study) is extremely difficult, even when using the feature selection methods proposed thus far.

Our deep learning model that uses the feature selection layer can evaluate ultra-high-dimensional features, and the importance of features can be learned by considering the complex interactions among the multiple features using dense layers. By inserting the feature selection layer immediately after the input layer, the input vector, which is multiplied by the weights of the feature selection layer and performs a nonlinear transformation, is regarded as the input to the subsequent network. The weights of the feature-selection layer are successively updated to increase the discrimination performance of the subsequent network through learning. Hence, the weights of the feature selection layer are considered to represent the importance of the input features, and features with higher weights are considered to be more effective in determining the discrimination capability of the later networks than features with lower weights, which do not contribute significantly to the performance of the network.

This study has several limitations. Because the number of samples used was very small (Task 1, 12; Task 2, 13), we could not prepare validation data when training the network, and we could not use external test data for evaluation. To improve the reliability of the output results, multiple trials were conducted, and further validation using TCGA database was performed. In addition, since the purpose of this study was to evaluate the feasibility of a new approach to discovering unknown genes related to cancer, we did not compare our method with other gene selection methods.

In conclusion, the use of our deep learning-based gene selection method for the selection of genes associated with pancreatic cancer development and prognosis was feasible. This method can be applied to gene expression data obtained for all diseases and may contribute to cancer stratification and to improving personalized medicine.

## Material and methods

### Study population criteria

The protocol was approved by the Institutional Review Board of Chiba Cancer Center (No. 28-15) and all patients and healthy volunteers provided their written informed consent. The study was carried out in accordance with the World Medical Association’s Declaration of Helsinki. In this study, 13 patients diagnosed with pancreatic ductal adenocarcinoma (PDAC) between January 2013 and December 2017 were included. All patients underwent surgery, and both specimens of cancer and frozen samples of nearby normal pancreatic tissue were collected. No preoperative chemotherapy was administered to any patient. Patients were observed for at least one year after surgery.

### RNA sequencing (RNA-seq)

Total RNA was extracted from frozen tissue blocks containing $50-100$ mg of PDAC tissue or normal tissue using standard protocols. First, frozen tissues were ground and homogenized using liquid nitrogen. Total RNA was extracted using an miRNeasy Mini Kit (QIAGEN) according to the manufacturer’s protocols. The quality, quantity, and integrity of total RNA were evaluated using a NanoDrop One/$\hbox {One}^{\mathrm {C}}$ UV-Vis spectrophotometer (Thermo Fisher Scientific) and a Bioanalyzer 2100 (Agilent Technologies). Only samples with an RNA quality score (RIN value)$> \, 7.0$ were used for RNA-seq. The RiboMinus Eukaryote System v2 was used to exclude rRNA from the total RNA. The mRNAs were barcoded with the Ion Xpress RNA-Seq Barcode 1–16 Kit (Thermo Fisher Scientific), and libraries were generated using the Ion Total RNA-Seq Kit v2 (Thermo Fisher Scientific). The library was constructed for next-generation sequencing (NGS) on an Ion Proton instrument (Thermo Fisher Scientific) using a $2\times 75$ bp pair-end protocol. In total, we sequenced eight libraries, generating 34–60 million pairs of reads per sample. NGS BAM files containing the sequence data were then analyzed by bioinformaticians. The number of reads mapped to annotated genomic features was quantified from the BAM files using the feature counts in the Subread package. The sample data were normalized to count per million (CPM).

### Deep learning-based gene selection

Deep learning can automatically extract the features needed for inference directly from raw data. However, the extracted feature information is distributed among the weights of the multilayered network, and it is usually difficult to obtain information about the basis of the inference in a form that humans can understand. We hypothesized that the addition of a feature selection layer in the deep learning model would solve this problem. The feature selection layer multiplies the input by weight in a one-to-one relationship and outputs vectors with the same shape as the input. Thus, the values of the input features are increased or decreased by the weights and passed to the next network.

A conceptual diagram of the feature-selection layer is shown in Fig. 1. When $\varvec{x}=(x_{1}, x_{2}, \cdots , x_{k})^{T}$, $\varvec{W}=(w_{1}, w_{2}, \cdots , w_{k})^{T}$, and $\varvec{y}=(y_{1}, y_{2}, \cdots , y_{k})^{T}$ are the input vector, the weights, and the output respectively, the operation performed by the feature selection layer is expressed as follows:

1 $$\begin{aligned} \varvec{y}= & {} f(\varvec{W}\otimes \varvec{x}), \end{aligned}$$

where $\otimes$ is the Hadamard product, and $f(\cdot )$ is the activation function. In the present study, a linear function of $f(x)=x$ was used as the activation function, and the value obtained after multiplication by the weights was directly passed to the network in the subsequent stage.

Figure 2 illustrates the network structure. The network after the feature selection layer comprised a very simple structure consisting of three 500-unit dense layers with a dropout probability of 0.5 and an output layer that outputs the probability of each class. The ReLU function was used as the activation function for all of the dense layers. Categorical cross-entropy was used as the loss function, and the weights of the feature-selection layer were regularized to be non-negative.

The gene expression data used in this study were based on the expression levels of 11,273 genes measured in 13 patients with pancreatic cancer. The gene expression data were performed the min-max normalization in order to present all values between 0 and 1. A feature selection layer with the same number of units as the genes was placed immediately after the input layer with the 11,273 units. The classification network was then placed behind it. Each layer in a neural network can be regarded as a kind of feature extractor. In other words, it can be thought of as a process that creates new features by applying complex nonlinear transformations from all the outputs of the previous layer. In particular, in the case of dense layers, the neurons in each layers consider the output of neurons in the previous layer as a new feature, and combine all of these features to produce a new feature. In general, neural networks are trained in such a way that the weights for features that contribute to reducing the loss function and the weights for unnecessary features are increased or decreased. Due to the min-max normalization for data and the non-negative regularization for feature selection layer, training proceeds in such a way that weights corresponding to features that are not necessary for minimizing the loss function are reduced. After model training, the weight of each unit in the feature selection layer is considered to represent the importance of the corresponding features.

Our method can be categorized as an embedding type of feature selection method. However, neural networks also include random factors, such as the initial values of weights and the order in which the training data are provided. In addition, when a neural network learns using high-dimension data from a low number of samples, the network may not be appropriately trained, and the weights of the feature selection layer may not be reliable. Thus, in this study, the importance of the genes was evaluated in terms of the number of times the genes were ranked in the top *N* positions after the network was trained *K* times. The ranking was sorted in descending order by weight corresponding to each feature in each training trial.

### Statistics

The differences between patients’ backgrounds were assessed using Fisher’s exact test and the Mann–Whitney U test. OS was defined as the period between surgery and final observation (in days), whereas DFS was defined as the time from surgery to tumor recurrence or death. A P-value of less than 0.05 was considered significant. These statistical analyses were conducted using JMP Pro version 13.2.0 (SAS Institute Inc., Cary, NC, USA). Survival analyses were performed using the R2: Kaplan Meier Scanner (http://r2.amc.nl) to separate the gene expression data of TCGA data into two groups and select the best Kaplan–Meier curves based on a log-rank test.
